# Serum Trimethylamine-*N*-Oxide Is Strongly Related to Renal Function and Predicts Outcome in Chronic Kidney Disease

**DOI:** 10.1371/journal.pone.0141738

**Published:** 2016-01-11

**Authors:** Catharina Missailidis, Jenny Hällqvist, Abdel Rashid Qureshi, Peter Barany, Olof Heimbürger, Bengt Lindholm, Peter Stenvinkel, Peter Bergman

**Affiliations:** 1 Department of Laboratory Medicine, Division of Clinical Microbiology, Karolinska University Hospital, Stockholm, Sweden; 2 Department of Forest Genetics and Plant Physiology, Swedish Metabolomics Centre, Swedish University of Agricultural Sciences, Umeå, Sweden; 3 Department of Clinical Science Intervention and Technology. Division of Renal Medicine, Karolinska University Hospital Huddinge, Stockholm, Sweden; University of Sao Paulo Medical School, BRAZIL

## Abstract

**Background:**

The microbial metabolite Trimethylamine-*N*-oxide (TMAO) has been linked to adverse cardiovascular outcome and mortality in the general population.

**Objective:**

To assess the contribution of TMAO to inflammation and mortality in chronic kidney disease (CKD) patients ranging from mild-moderate to end-stage disease and 1) associations with glomerular filtration rate (GFR) 2) effect of dialysis and renal transplantation (Rtx) 3) association with inflammatory biomarkers and 4) its predictive value for all-cause mortality.

**Methods:**

Levels of metabolites were quantified by a novel liquid chromatography/tandem mass spectrometry-based method in fasting plasma samples from 80 controls and 179 CKD 3–5 patients. Comorbidities, nutritional status, biomarkers of inflammation and GFR were assessed.

**Results:**

GFR was the dominant variable affecting TMAO (β = -0.41; p<0.001), choline (β = -0.38; p<0.001), and betaine (β = 0.45; p<0.001) levels. A longitudinal study of 74 CKD 5 patients starting renal replacement therapy demonstrated that whereas dialysis treatment did not affect TMAO, Rtx reduced levels of TMAO to that of controls (p<0.001). Following Rtx choline and betaine levels continued to increase. In CKD 3–5, TMAO levels were associated with IL-6 (Rho = 0.42; p<0.0001), fibrinogen (Rho = 0.43; p<0.0001) and hsCRP (Rho = 0.17; p = 0.022). Higher TMAO levels were associated with an increased risk for all-cause mortality that remained significant after multivariate adjustment (HR 4.32, 95% CI 1.32–14.2; p = 0.016).

**Conclusion:**

Elevated TMAO levels are strongly associated with degree of renal function in CKD and normalize after renal transplantation. TMAO levels correlates with increased systemic inflammation and is an independent predictor of mortality in CKD 3–5 patients.

## Introduction

The phenotype of CKD is often accompanied by systemic inflammation and oxidative stress, which promote progression of CKD, premature aging [1] and cardiovascular disease (CVD) [2–6]. Thus, the prospects for this high-risk patient group remain somber. Lately, the gut as a contributing factor in the systemic inflammatory response observed in CKD has been in focus. Evidence suggest that uremia induced impairment of the epithelial barrier function together with compositional changes in the gut microbiota [7–11] enables translocation of endotoxins and microbial metabolites enhancing systemic inflammation [10–13]. Moreover, colonic bacteria are the main producers of pro-inflammatory uremic toxins such as indoxyl sulfate and p-cresyl sulfate [10, 12].

TMAO is a gut-derived metabolite that has been linked to CVD and mortality in both humans and in animal models [14–17]. TMAO is generated by bacterial conversion of phosphatidylcholine, choline, betaine and carnitine, into gaseous trimethylamine [14, 15, 18] that is taken up and oxidized into TMAO by flavin-containing monooxygenases (FMO1 and FMO3) in the liver [19]. Dietary sources of TMAO include meat, egg, dairy products and salt water fish [14, 18, 20]. Although diet may influence levels of TMAO and its metabolic precursors, evidence suggests that the microbial composition of gut flora is the major contributing factor in regulating circulating TMAO levels [14, 20]. Other factors governing TMAO levels are FMO enzyme activity and renal clearance; decreased renal function has been linked with elevated TMAO levels that were effectively cleared by dialysis [21, 22].

The mechanism by which TMAO promotes atherosclerosis and increases cardiovascular risk is not completely understood. TMAO has been linked to macrophage activation, foam cell formation and altered cholesterol metabolism in animal studies [14, 17]. Although several reports demonstrate an association between higher TMAO levels and CVD [14, 17] and heart failure [16, 23], little has yet been published on how the metabolite is associated with known inflammatory and pro-coagulant risk markers for CVD. Furthermore, the contribution of TMAO on inflammation and premature mortality in CKD still remains to be elucidated. Whereas two recent studies demonstrated that increased levels of TMAO in mild-moderate CKD associated with coronary artery disease pathogenesis and mortality [24, 25], another study on prevalent dialysis patients reported no effect on mortality [26].

Considering the increased levels of TMAO observed in CKD, it is reasonable to hypothesize that TMAO may act as a gut-derived uremic toxin contributing to systemic inflammation and in extension CVD and premature mortality. In this study, we present data on TMAO and related metabolites in carefully phenotyped patients with CKD stage 3–5. We examined levels of TMAO and 1) associations with renal function 2) effect of dialysis and Rtx 3) association with inflammatory biomarkers and 4) its predictive value for all-cause mortality.

## Methods

### Patients and study design

Fasting plasma samples from CKD 3–4 and CKD 5 patients, consecutively recruited into two observational prospective cohort studies previously described [3, 5, 27], at Karolinska University Hospital, Stockholm, Sweden, were analyzed for TMAO, choline, betaine and systemic markers of inflammation, metabolism and renal function. Comorbidities and nutritional status were assessed based on medical records, and subjective global assessment (SGA) score was used as a surrogate marker of protein-energy wasting (PEW) [28]. A majority of the patients were Caucasians; however, ethnic background was not registered. Survival was recorded from date of inclusion with a follow up of five years, or up to the time of Rtx. No patient was lost to follow-up. Exclusion criteria were age <18 years, active hepatitis B/C, HIV and signs of acute infection. The Ethics Committee of the Karolinska Institutet approved the study protocol and informed written consent was obtained from all patients.

Controls (*n* = 80) consisted of age- and sex-matched population-based individuals in the Stockholm region of Sweden, randomly selected by Statistics Bureau of Sweden (www.scb.se). No other exclusion criteria than unwillingness to participate in the study were applied in the selection of the healthy controls

CKD 3–4 patients (*n* = 58) were included during 2001–2008 with an equal distribution between CKD 3 (*n* = 30) and CKD 4 (*n* = 28) according to measured GFR.

CKD 5 patients (n = 116) were included during 2000–2012, close to start of dialysis treatment. After inclusion 33% were subsequently treated with hemodialysis (HD) and 67% with peritoneal dialysis (PD). As expected, the majority were on antihypertensive medications as well as other commonly used drugs in CKD, such as phosphate-binders, diuretics and vitamins B, C and D supplementation.

A subset (*n* = 74) of the CKD 5 patients (61% males, mean age 53±12 years, mean BMI 25±4 kg/m^2^, 18% smokers, 16% diabetes) were followed from inclusion and reassessed after 12 months of dialysis treatment and/or 12 months and 24 months after Rtx, respectively. Whereas 25% were treated with HD and 65% with PD before Rtx, 10% underwent pre-emptive Rtx

### Estimation of nutritional status

SGA was used to evaluate PEW as previously described [28]. SGA scoring included six subjective assessments reflecting nutritional status. Three of the assessments were based on the patient’s history of weight loss, incidence of anorexia and vomiting and three were based on subjective grading of muscle wasting, presence of oedema and loss of subcutaneous fat.

### Analysis of TMAO, choline and betaine in human plasma

Plasma heparin samples were obtained after a 12 h fast and stored at -80°C. Quantification of TMAO, choline and betaine was performed by LC-MS/MS, utilizing a protocol designed specifically for this purpose and prepared in a 96-well format.

Extracted plasma aliquots were spiked with internal standards, comprised of TMAO-D_9_ in methanol and water with Proline-^13^C_5_ as a recovery standard, and injected on an Agilent 1290 Infinity chromatographic system (Agilent Technologies, Waldbronn, Germany) fitted with an Acquity UPLC Amide column in combination with a VanGuard precolumn (Waters Corporation, Milford, MA, USA).

The compounds were detected with an Agilent 6490 Triple Quadrupole mass spectrometer (Agilent Techologies, Santa Clara, CA, USA). Data processing was performed with MassHunter Quantitative Analysis QQQ (Agilent Technologies Inc. Santa Clara, CA, USA).The MS/MS analysis for TMAO, choline, betaine, TMAO- D_9_ and Proline-^13^C_5,_ were conducted in multiple-reaction-monitoring (MRM) mode at *m/z* 76→58, *m/z* 104→45, *m/z* 118→58, *m/z* 85→66 and *m/z* 121→74 respectively.

The TMAO assay was linear up to 0.25 ng injected on column (S1 Fig). The method demonstrated low intraday and interday coefficients of variance (CV) of < 4.86 and 2.21% respectively (S1 Table). Metabolite levels remained stable and comparable in assessment of plasma heparin and serum samples and multiple number of freeze-thaw cycles (S2 Fig).

### Analysis of markers of systemic inflammation, metabolism and renal function

Plasma levels of IL-6 were measured by a commercially available photometric enzyme-linked immunosorbent assays (ELISA) (Boehringer Mannheim, Mannheim, Germany). Circulating levels of albumin, creatinine, calcium, phosphatase, hemoglobin, fibrinogen and hsCRP were analyzed according to certified methods at the Karolinska University Laboratory, Unit of Clinical Chemistry, Karolinska University Hospital, Sweden.

Measured GFR (mGFR), which represents a more accurate estimation of renal function, was measured by Iohexol clearance in controls and CKD 3–4 patients. In CKD 5 patients, mGFR was calculated by the mean of renal urea and creatinine clearance from a 24-hour urine collection. Estimated glomerular filtration rate (eGFR) used in follow-up after Rtx was calculated by a cystatin C-based equation for estimation of GFR; 130 x cystatin C -1,069 x age -0,117–7, as described previously [29].

### Statistical analysis

Data are expressed as mean ± standard deviation or median (10th to 90th percentile) or percentage or hazard ratio (HR; 95% confidence intervals (CI)), as appropriate. Statistical significance was set at the level of P <0.05. Wilcoxon’s rank-sum test performed comparisons between two groups. Comparisons of nominal variables between groups were made by Fischer’s exact test. Comparisons between >2 groups were assessed with nonparametric analysis of variance. Spearman’s correlation was used to determine correlations between variables. We used multiple linear regression analysis to assess determinants of TMAO, choline and betaine levels (adjusting for age, gender, SGA, albumin, DM and mGFR) and inflammation (adjusting for age, gender, smoking, mGFR and metabolites). To study the impact on the clinical outcome, a Kaplan-Meier survival curve and multivariable PROC PHREG regression analysis was performed. Age, gender, DM, hsCRP and mGFR, were included in the model. Statistical analyses were performed using statistical software SAS version 9.4 (SAS Campus Drive, Cary, NC, USA) and GraphPad prism 5 (7825 Fay Avenue, Suite 230 La Jolla, CA 92037 USA).

## Results

### Baseline characteristics of CKD patients and healthy controls

The basal clinical and laboratory characteristics of the CKD 3–4 and CKD 5 patients and controls are summarized in Table 1. The cohorts were comparable in terms of age, gender and BMI. As expected, CKD 5 patients had a higher burden of comorbidities, including diabetes mellitus, CVD and PEW, lower mGFR, plasma albumin and hemoglobin levels and higher levels of creatinine, hsCRP and IL-6, compared with CKD 3–4 patients and controls.

**Table 1:** Demographic and laboratory characteristics of controls and CKD patients.

	Controls (*n* = 80)	CKD 3–4 (*n* = 63)	CKD 5 (*n* = 116)	Total CKD cohort (CKD 3–5, *n* = 179)
Age, years	62 ± 12	56 ± 16	55 ± 13	55 ± 14
Male gender, n (%)	57 (71)	42 (72)	74 (61)	116 (65)
BMI, kg/m^2^	26 ± 4	27 ± 5	25 ± 5	25 ± 5
Smoking*, n (%)	16 (22)	4 (2)	16 (20)	20 (16)
Diabetes*, n (%)	4 (5)	11 (22)	33 (27)	44 (26)
CVD, n (%)	10 (13)	13 (21)	38 (33)	51 (28)
Cause of kidney disease, n (%):				
Polycystic kidney disease		9 (15)	21 (18)	30 (17)
Nephrosclerosis		6 (10)	6 (5)	12 (7)
Diabetic nephropathy		5 (8)	30 (26)	35 (20)
Glomerulonephritis		21 (34)	27 (23)	48 (27)
Unknown or other aetiology		22 (34)	32 (28)	54 (30)
SGA >1*, n (%)	2 (2.5)	1 (0.7)	25 (23)	26 (16)
mGFR, mL/min	83 (68–104)	28 (19–45)	7 (4–10)	9 (5–36)
Creatinine, μmol/L	79 (60–98)	206 (142–366)	714 (448–1047)	576 (177–979)
Albumin, g/L	39 (36–43)	38 (23–42)	32 (27–39)	36 (28–40)
Calcium, mmol/L	2.3 (2.2–2.4)	2.3 (2.2–2.6)	2.4 (2.1–2.7)	2.4 (2.1–2.7)
Phosphate, mmol/L	1.0 (0.8–1.2)	1.1 (0.9–1.6)	2.0 (1.4–2.7)	1.7 (1.1–2.52)
Hemoglobin, g/L	144 (130–156)	128 (112–147)	110 (94.0–126)	114 (97–136)
hsCRP, mg/L	1.3 (0.4–7.0)	2.5 (0.5–8.50)	3.3 (0.6–25.1)	2.8 (0.6–16.8)
IL-6, pg/mL		2.0 (1.5–5.8)	3.0 (2.1–15.6)	3.7 (1.9–11.9)
Fibrinogen, g/L	2.9 (2.4–3.9)	3.7 (2.8–4.9)	4.6 (3.3–6.6)	4.2 (2.9–6.30)
TMAO, μM/L	5.8 (3.1–13.3)	14.6 (5.6–71.2)	73.5 (26.4–191.0)	53.4 (9.3–170.0)
Choline, μM/L	66.5 (55.9–81.3)	65.0 (44.4–95.8)	77.2 (48.0–140.0)	71.6 (47.0–122.0)
Betaine, μM/L	92.2 (66.4–130.0)	60.5 (37.9–93.1)	21.5 (10.5–52.1)	40.5 (14.6–81.9)

**Abbreviations and definitions**: BMI, body mass index; CVD, Cardiovascular disease, defined as cerebrovascular (including stroke), cardiac or peripheral disease;.SGA, subjective global assessment with score > 1 indicating presence of protein-energy wasting; mGFR, measured glomerular filtration rate.

All values are expressed as the mean ± SD, median (10th -90th percentile), or number (%) as appropriate.

* Values missing in all cohorts.

### Renal function was a major determinant of TMAO-levels

**Fig 1:**
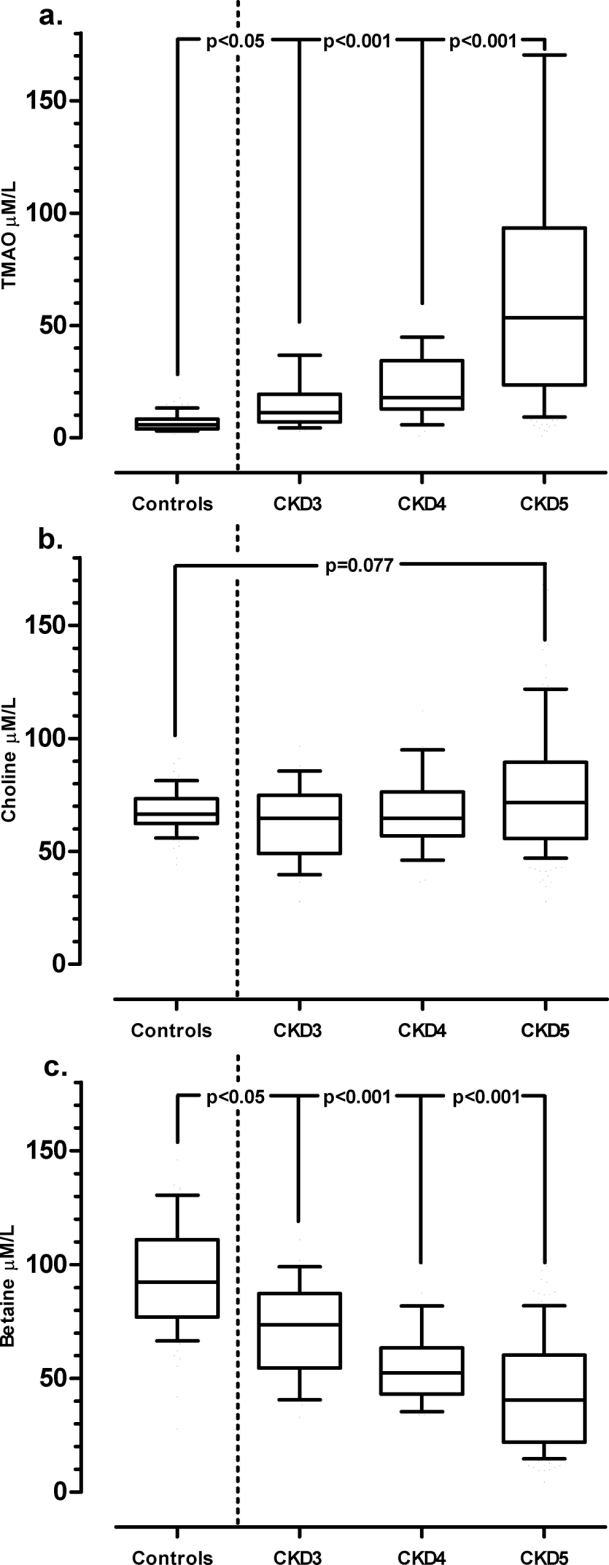
Increasing plasma levels of TMAO in CKD patients stage 3 (*n* = 30), stage 4 (*n* = 28) and stage 5 (*n* = 116) compared to healthy controls (*n* = 80). Values are expressed as median (10^th^ -90^th^ percentile). P-values analyzed by Kruskal–Wallis’ one-way ANOVA, followed by Dunn’s multiple comparison test.

CKD patients had higher TMAO levels than controls and the levels rose with decreasing renal function (Fig 1A). Consequently, CKD 5 patients had a 13-fold increase in TMAO compared to controls (Fig 1A). Levels of choline did not differ significantly between CKD stages and controls (Fig 1B). In contrast, betaine levels decreased with each CKD stage (Fig 1C) and CKD 5 patients had 4-fold lower level of betaine compared to controls (Fig 1C).

In CKD 3–4 patients, levels of TMAO (Rho = -0.15; p<0.0001) and choline (Rho = -0.31; p = 0.023) correlated inversely with mGFR, whereas betaine correlated with higher mGFR (Rho = 0.33; p = 0.015). In CKD 5 patients, TMAO exhibited a similar trend (Rho = -0.20; p = 0.062), whereas the association was lost for the other metabolites, possibly due to the narrow range of mGFR in this group of patients. When analyzing the total CKD cohort (CKD 3–5, n = 179), TMAO (Rho = -0.69; p<0.0001) and choline (Rho = -0.32; p<0.0001) demonstrated a robust inverse correlation with mGFR (S3 Fig), whereas betaine (Rho = 0.58; p<0.0001) correlated positively with mGFR (S3 Fig). In multiple regression analysis of potential determinants for the metabolites, chosen to reflect nutritional status (SGA>1, plasma albumin), DM and renal function in the total CKD cohort, mGFR was the most dominant variable for TMAO (β = -0.41, p<0.001), choline (β = -0.38, p<0.001), and betaine (β = 0.45, p<0.001) levels, respectively (Table 2).

**Table 2:** Multiple regression models of determinants for plasma TMAO, choline and betaine in total CKD cohort (CKD 3–5, *n* = 179).

	TMAO Mode (β, *P*) (r^2^ = 0.30)	Choline Model(β, *P*) (r^2^ = 0.18)	Betaine Model (β, *P*) (r^2^ = 0.25)
Age	(-0.018, 0.793)	(-0.017, 0.816)	(-0.004, 0.960)
Gender	(0.152, 0.028)	(0.063, 0.393)	(0.078, 0.268)
SGA >1	(-0.131, 0.070)	(-0.163, 0.037)	(-0.077, 0.299)
Albumin	(-0.275, <0.001)	(-0.142, 0.079)	(0.019, 0.805)
DM	(0.003, 0.966)	(0.019, 0.804)	(0.017, 0.808)
mGFR	(-0.414, <0.001)	(-0.378, <0.001)	(0.452, <0.001)

**Abbreviations and definitions**: SGA, subjective global assessment with score > 1 indicating presence of protein-energy wasting; DM, diabetes mellitus; mGFR, measured glomerular filtration rate.

### Effects of dialysis and renal transplantation on metabolites

**Fig 2:**
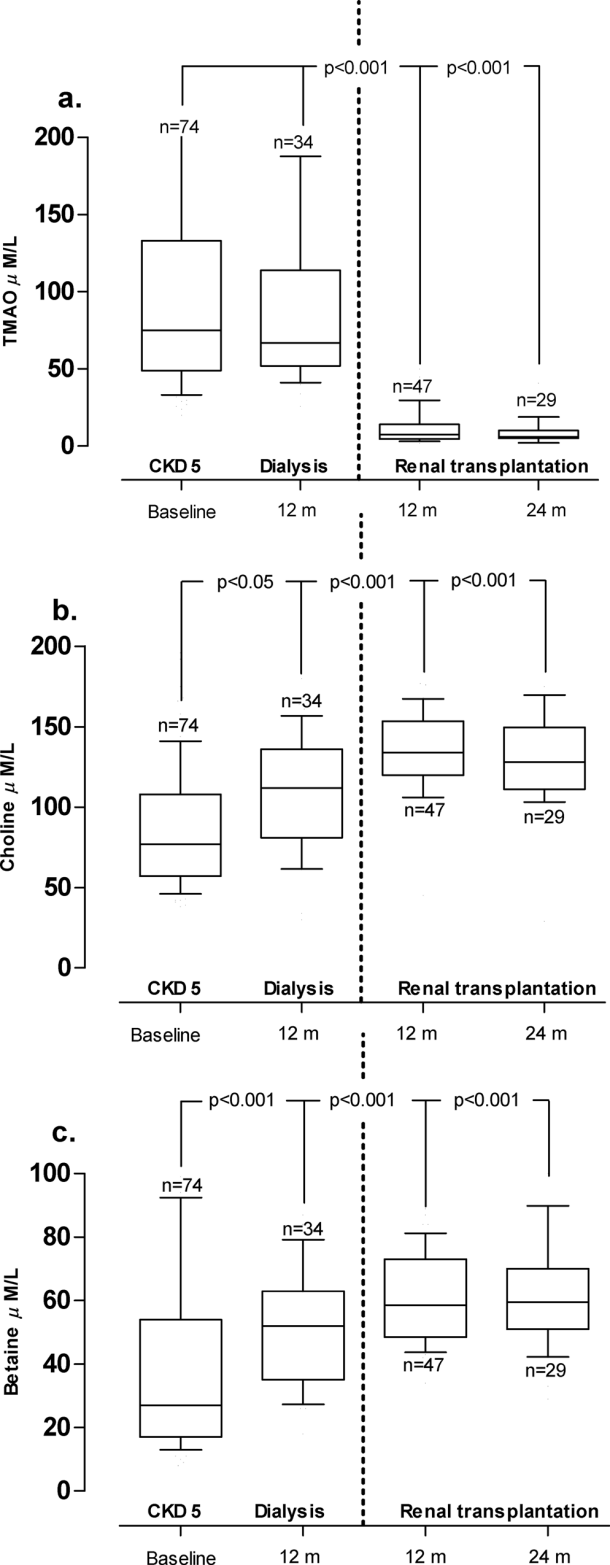
Comparison of plasma levels of metabolites in a cohort of CKD 5 patients (*n* = 74) followed from baseline and reassessed 12 months after start of dialysis and/or 12 months and 24 months after renal transplantation (Rtx). Choline and betaine levels increased with dialysis and Rtx whereas TMAO levels remained unchanged during dialysis and normalized after Rtx. Due to missing samples box-plots represents 34/74 patients at 12 months of dialysis, 47/74 patients at 12 months and 29/74 patients at 24 months follow-up after Rtx. Values are expressed as median (10^th^ -90^th^ percentile) P-values analyzed by Kruskal–Wallis’ one-way ANOVA, followed by Dunn’s multiple comparison test.

As expected, Rtx led to a significant improvement in eGFR (p<0.0001) (Table 3), improved nutritional status, and increased plasma albumin levels (Table 3). Whereas baseline TMAO levels remained unchanged after 12 months of dialysis treatment, it decreased dramatically after Rtx (p<0.001) (Fig 2A). In contrast, choline and betaine levels increased significantly after 12 months of dialysis (p<0.01 and p<0.001, respectively) (Fig 2B and 2C) and continued to rise after Rtx (Fig 2B and 2C). PD or HD treatment did not have a significant impact on the levels of metabolites at any time-point.

**Table 3:** Plasma levels of metabolites, renal function and nutritional assessment in CKD 5 patients followed from baseline and reassessed after 12 months of dialysis treatment and/or 12 months and 24 months after renal transplantation (Rtx).

	Baseline (*n* = 74)*	12 months dialysis	12 months Rtx.	24 months Rtx.	*P* value
SGA >1, n (%)	13 (19)	5 (17)	0 (0)	0 (0)	0.0028
Albumin, g/L	35 (29–39)	34 (32–36)	37 (36–38)	37 (36–38)	<0.0001
Creatinine, μmol/L	774 (467–1121)		120 (109–132)	119 (103–136)	<0.0001
eGFR, mL/min/1.73m^2^	11 (9–13)		50 (28–76)	54 (28–78)	<0.0001
hsCRP, mg/L	1.9 (0.5–13.8)	5.4 (0.9–34)	1.7 (0.4–4.7)	1.3 (0.4–9.4)	0.0015
TMAO, μM/L	74.5 (34.2–192.0)	69.6 (42.1–198.0)	6.9 (2.6–24.7)	5.5 (2.0–19.4)	<0.0001
Choline, μM/L	77.5 (50.2–155.0)	112.0 (61.8–157.0)	134.0 (107.0–167.0)	128 (102.0–171.0)	<0.0001
Betaine, μM/L	22.3 (11.3–53.5)	53.8 (26.8–79.7)	59.6 (43.4–80.4)	61.1 (42.4–90.3)	<0.0001

*74 CKD 5 patients where followed longitudinally through dialysis to renal transplantation. Due to missing samples data table represents 34/74 patients at 12 months of dialysis, 47/74 patients at 12 months and 29/74 patients at 24 months follow up after renal transplantation.

**Abbreviations and definitions**: Rtx, renal transplantation; SGA, subjective global assessment with score > 1 indicating presence of protein-energy wasting; Alb, albumin; eGFR, estimated glomerular filtration rate assessed by cystatin C clearance. Values represented as number (percentage) and median (10^th^ -90^th^ percentile). P-values analyzed by Chi square test and Kruskal–Wallis’ one-way ANOVA, followed by Dunn’s multiple comparison test.

### Microbial metabolites in relation to inflammation biomarkers

**Fig 3:**
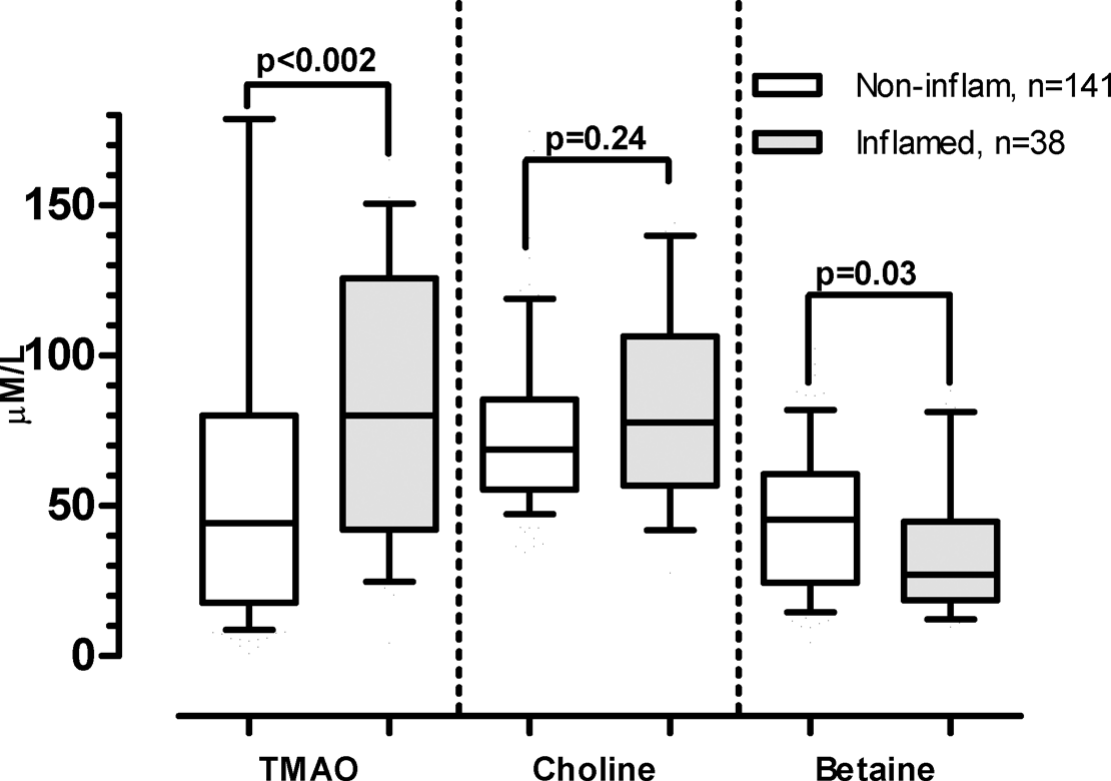
Comparative analysis of metabolites in inflamed (hsCRP ≥10 mg/L) and non-inflamed (hsCRP < 10 mg/L) CKD 3–5 patients (*n* = 179). Higher hsCRP levels associated with higher TMAO levels and decreased betaine levels. Values are expressed as median (10^th^ -90^th^ percentile). P-values analyzed by Kruskal–Wallis’ one-way ANOVA, followed by Dunn’s multiple comparison test.

When analyzing the total CKD cohort, correlations were observed between TMAO and IL-6 (Rho = 0.42; p<0.0001) and fibrinogen (Rho = 0.43; p<0.0001) (S3 Fig). A similar trend was observed for choline and IL-6 (Rho = 0.26; p = 0.007) (S3 Fig), but not for choline and fibrinogen (S3 Fig). Betaine levels were inversely correlated with both IL-6 (Rho = -0.21; p = 0.029) and fibrinogen (Rho -0.34; p<0.0001) (S3 Fig). A weak, but significant, correlation was observed between TMAO and hsCRP (Rho = 0.17; p = 0.022) (S3 Fig), but not for choline or betaine. Comparative analysis of inflamed (hsCRP ≥ 10 mg/L) and non-inflamed patients (hsCRP < 10 mg/L), demonstrated higher TMAO levels (p<0.002) and lower betaine levels (p = 0.031) in inflamed patients **(**Fig 3).

In regression analysis assessing the effect of metabolites and mGFR on degree of inflammation in the total CKD cohort, GFR was the dominant variable in the TMAO and betaine models. Only betaine and fibrinogen maintained a significant association (β = -0.153, p *=* 0.015, r^2^ = 0.54), whereas TMAO and choline had no significant effect on the estimate for IL-6, fibrinogen or hsCRP when GFR was taken into account.

### Elevated TMAO levels were associated with reduced 5-year survival

**Fig 4:**
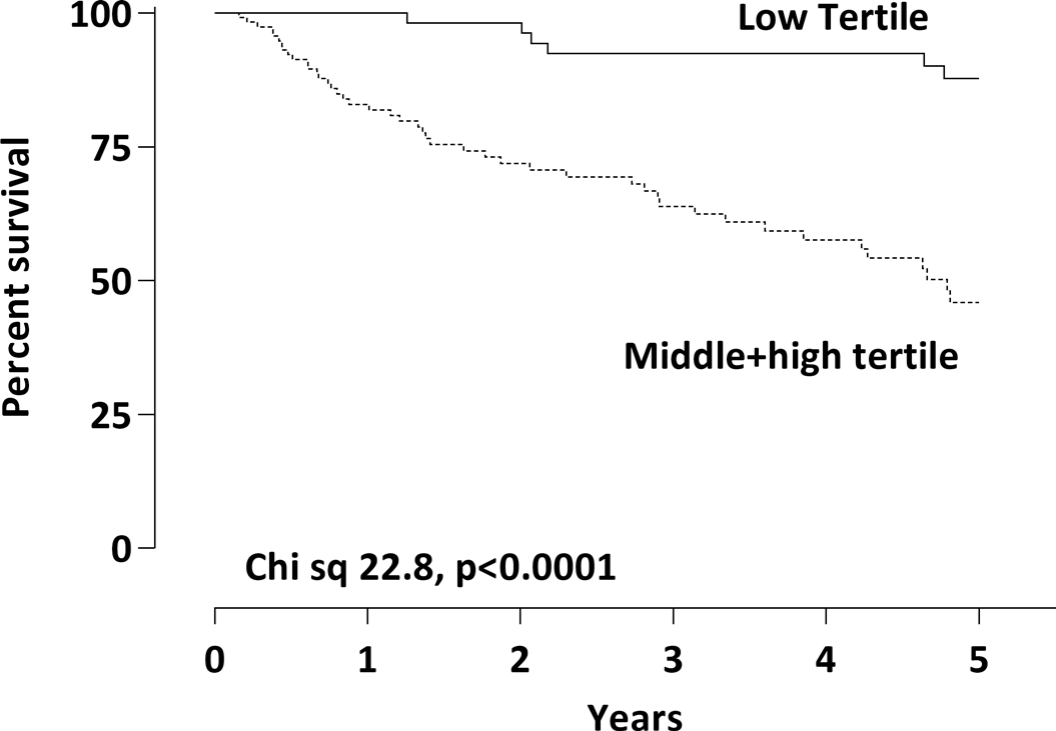
Kaplan-Meier analysis of TMAO levels and all-cause mortality in CKD 3–5 patient (*n* = 179). Data presented as tertiles. CKD patients with the highest TMAO levels (Combined middle (32.2–75.2 μM/L) + high tertile (>72.2 μM/L)) had a significantly lower survival compared with patients in the lowest TMAO tertile.

During five years follow-up, a total of 51 (28%) patients died and 88 (49%) underwent Rtx. Kaplan-Meier analysis of metabolites divided in tertiles revealed that levels of TMAO, but not choline or betaine, were associated with decreased survival. CKD patients with the highest TMAO levels (Combined middle; 32.2–75.2 μM/L+ high tertile; >72.2 μM/L) had a significantly lower survival compared with patients in the lowest TMAO tertile (<32.2 μM/L) (Chi square 22.8, p<0.0001) (Fig 4). In unadjusted Cox-regression analysis higher TMAO levels were associated with a 6.3-fold risk increase for all-cause mortality. This association was attenuated, but remained significant, following stepwise adjustment for gender, age diabetes, hsCRP and mGFR (HR 4.32, 95% CI 1.32–14.2, p = 0.016) (Table 4).

**Table 4:** Cox proportional hazards analysis of plasma TMAO levels stratified by tertiles in predicting risk of all-cause mortality at 5 years in total CKD cohort (CKD 3–5 patients, *n* = 179).

Variable	HR (95% CI)	*P*-value
Middle (32.2–75.2 μM/L)+ high tertile (>72.2 μM/L)	6.29 (2.67–14.8)	<0.0001
+gender+age	6.16 (2.59–14.7)	<0.0001
+gender+age+DM	8.23 (2.90–23.4)	<0.0001
+gender+age+DM+hsCRP	6.68 (2.33–19.1)	0.0004
+gender+age+DM+hsCRP+GFR	4.32 (1.32–14.2)	0.016

**Abbreviations**: DM, diabetes mellitus; hsCRP, high sensitivity CRP; GFR, glomerular filtration rate

## Discussion

The present study show that impaired renal function is a major determinant of plasma levels of TMAO in CKD. Moreover, TMAO levels were associated with increased levels of systemic inflammatory biomarkers, but the association was lost when controlling for mGFR. Finally, high levels of TMAO predicted reduced 5-year survival that remained significant after multivariate adjustment.

The finding that TMAO levels are elevated in CKD and inversely associate with GFR has been observed in several studies [15, 16, 19, 22, 25]. The observation that TMAO levels decline rapidly after Rtx and remain low after two years corroborates recent findings in 6 patients by Stubbs *et al* [25]. However, the distribution and inflammatory associations of the TMAO precursors, choline and betaine in CKD have not been previously described.

Our results suggest that dietary recommendations may affect levels of choline and betaine in CKD but have less impact on TMAO levels. A protein-restricted diet -commonly prescribed to CKD 3–5 patients—corresponds with the unchanged choline and decreasing betaine levels observed with each CKD stage. Notably, levels of choline and betaine increased after 12 months of dialysis, which may reflect guideline recommendations of increased protein intake for dialysis patients [30]. These presumably dietary related changes did not, however, result in the corresponding increases in TMAO levels that have been shown in previous studies [15, 17]. Instead, TMAO-levels remained unchanged after 12 months of dialysis. It is intriguing that whereas Rtx dramatically reduced TMAO to control levels, choline and betaine levels increased even further. Based on these observations we draw two conclusions: First, dietary changes could not explain the normalized levels of TMAO after Rtx, since levels of the TMAO precursors, betaine and choline, increased. Second, the reduction of TMAO levels after Rtx, reinforce that decreased renal clearance is the major cause of elevated TMAO levels in the uremic milieu.

Interestingly, and in contrast to previous studies, we found strong associations between TMAO and inflammatory biomarkers. We also observed similar but inverse associations for betaine and inflammatory biomarkers. In accordance, Troseid *et al*. [16] reported that whereas increased LPS levels in 155 patients with chronic heart failure did not associate with TMAO or choline it was inversely associated with betaine levels. In contrast Srinivasa *et al* [31] studied TMAO, choline and betaine in HIV patients and reported no association with IL-6, hsCRP or LPS. However, these patients did not differ from controls regarding levels of metabolites and inflammatory markers, which possibly could explain the lack of associations. Tang *et al*. [24] found no association between TMAO and hsCRP in 521 CKD 3 patients, whereas Kaysen *et al*. [26] reported a paradoxical inverse association between TMAO and hsCRP in 235 prevalent HD patients. The reason for the different outcome in these two CKD population compared to our study is not clear. However, the study population of Tang *et al*. had ten-fold higher levels of hsCRP than reported by Kaysen *et al*. and our study, suggesting active inflammatory events that could affect the outcome. On the other hand, differences in TMAO metabolism and inflammation between prevalent dialysis patients and CKD 5 patients not yet on dialysis treatment as in our study could explain the different outcome reported by Kaysen *et al*. [26].

Although the significant associations between TMAO and inflammatory markers were lost when renal function was accounted for, suggesting that TMAO might be a surrogate marker for GFR, we cannot exclude that TMAO may have direct proinflammatory and even nephrotoxic properties. Tang *et al*. [24] demonstrated that dietary induced TMAO elevation directly contributed to progressive renal fibrosis and dysfunction in mice. In further support of this notion, a metabolite profiling of participants in the Framingham Heart Study, identified plasma TMAO and choline as markers predicative of CKD at 8 years follow-up that were not correlated with baseline eGFR [32].

In accordance with previous studies on patients with CVD [14, 17], heart failure [16, 23] and CKD [24, 25] we found that raised TMAO levels predicted a higher 5-year mortality rate. The significant association between TMAO and outcome remained after multivariate adjustments, suggesting that TMAO may be an independent risk factor for mortality in CKD 3–5. However, as the significance was reduced when controlling for hsCRP and mGFR it is possible that the dominance of incident dialysis patients, who represented 68% of the investigated patients, may have affected the outcome. It is described that dialysis patients have a considerably higher mortality risk than CKD patients [33]. Moreover, the relatively small study sample size prohibited extensive multivariate regression analysis thus limiting the ability to draw firm conclusions.

The present study has several strengths: i) it is the first study presenting an in-depth analysis of relations between renal function and TMAO levels, and its dietary precursors, in two groups of well-defined and extensively characterized patients with CKD ranging from mild-moderate (CKD 3–4) to advanced (CKD 5) stage; ii) it presents longitudinal data on the interventional effects of dialysis and Rtx on TMAO levels in CKD 5 patients; iii) the CKD 3–4 and CKD 5 cohorts were relatively homogeneous in terms of age, sex, and etiology of CKD, which lends additional credibility to our results; iv) we also had access to a control group of age and sex-matched, but otherwise randomly selected individuals from a community-based population. Some limitations, other than the above mentioned, should be acknowledged; i) the observational nature of the study precludes any inferences regarding causality and finally; ii) the lack of CKD stage 1–2 patients may limit the generalizability of the results.

In summary, we report that elevated TMAO levels are strongly associated with degree of renal function in CKD and normalize after Rtx. TMAO levels correlates with increased systemic inflammation and is an independent predictor of mortality in this cohort of CKD 3–5 patients.

## Supporting Information

S1 Fig Standard curve for analysis of TMAO concentration. Standard samples were prepared by adding 20 μL of blank samples extracted with TMAO-d_9_ (TMAO-d_9_ concentration in extract = 0.1 ng/μL) to micro vials. 50 μL standard solutions of different concentration (rendering the range of 0.0005–0.25 ng on column) were then added to and dried whereupon the standard samples were re-dissolved in 20 μL methanol and 20 μL water containing the recovery standard Proline-^13^C_5_ (Proline-^13^C_5_ concentration in water = 2 ng/μL).
(PDF)

S2 Fig Principal component analysis (PCA) score plot on how multiple freeze-thaw cycles and choice of serum or plasma samples from one person affects the ratio of analyte/internal standard response. Labels show numbers of freeze-thaw cycles. Data points denote whether it is a serum sample (■gel and▼red) or a plasma sample (○EDTA and ▲heparin)
(PDF)

S3 Fig Linear regression analysis depicting the relationship between metabolites and mGFR in total CKD cohort (n = 179) (PDF)

S4 Fig Linear regression analysis depicting the relationship between metabolites and the inflammatory biomarkers; IL-6, fibrinogen and hsCRP in total CKD cohort (n = 179). (PDF)

S1 Table Coefficients of variation (CV) and precision of TMAO concentrations. (PDF)
