# Supplementary figures and images for: Serum Trimethylamine-N-Oxide Is Strongly Related to Renal Function and Predicts Outcome in Chronic Kidney Disease
Source: PLoS One. 2016 Jan 11;11(1):e0141738. doi: 10.1371/journal.pone.0141738 (PMC4709190; doi:10.1371/journal.pone.0141738)

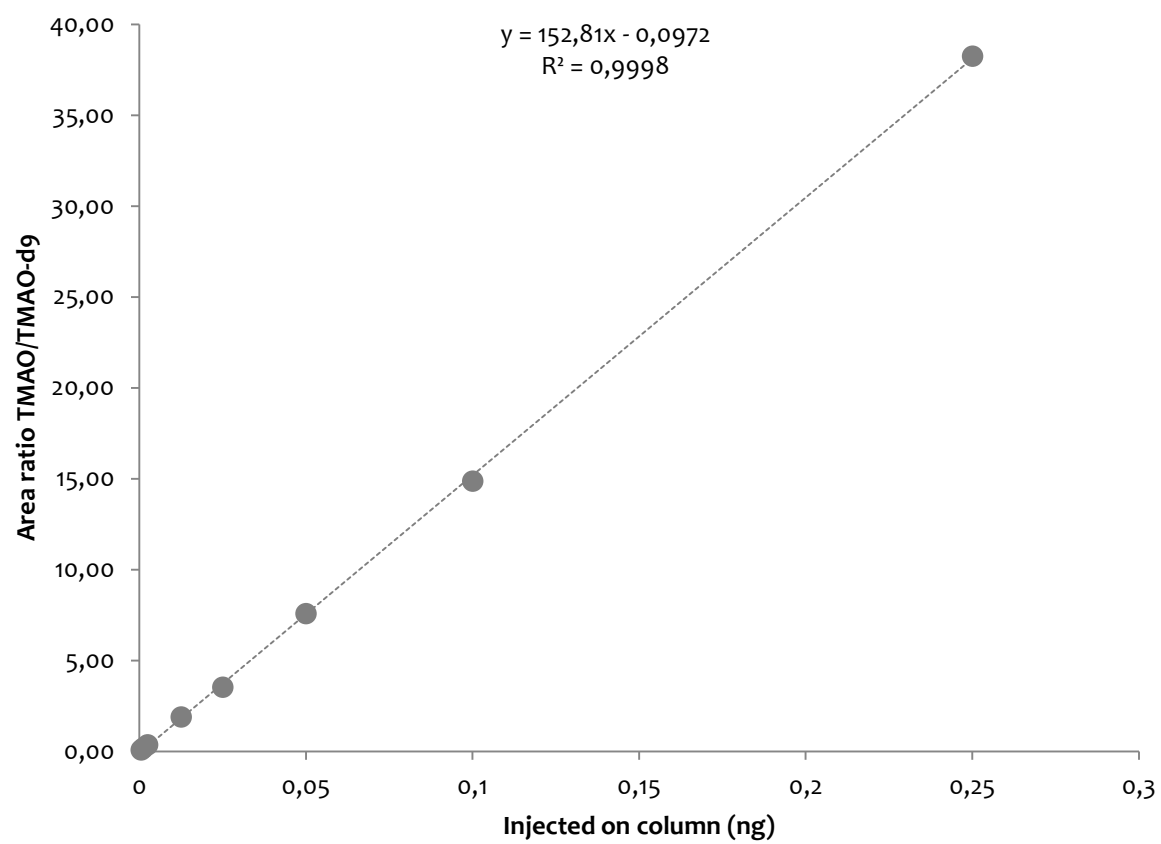

Supplement: S1 Fig — Standard samples were prepared by adding 20 μL of blank samples extracted with TMAO-d9 (TMAO-d9 concentration in extract = 0.1 ng/μL) to micro vials. 50 μL standard solutions of different concentration (rendering the range of 0.0005–0.25 ng on column) were then added to and dried whereupon the standard samples were re-dissolved in 20 μL methanol and 20 μL water containing the recovery standard Proline-13C5 (Proline-13C5 concentration in water = 2 ng/μL). (PDF) [file pone.0141738.s001.pdf]

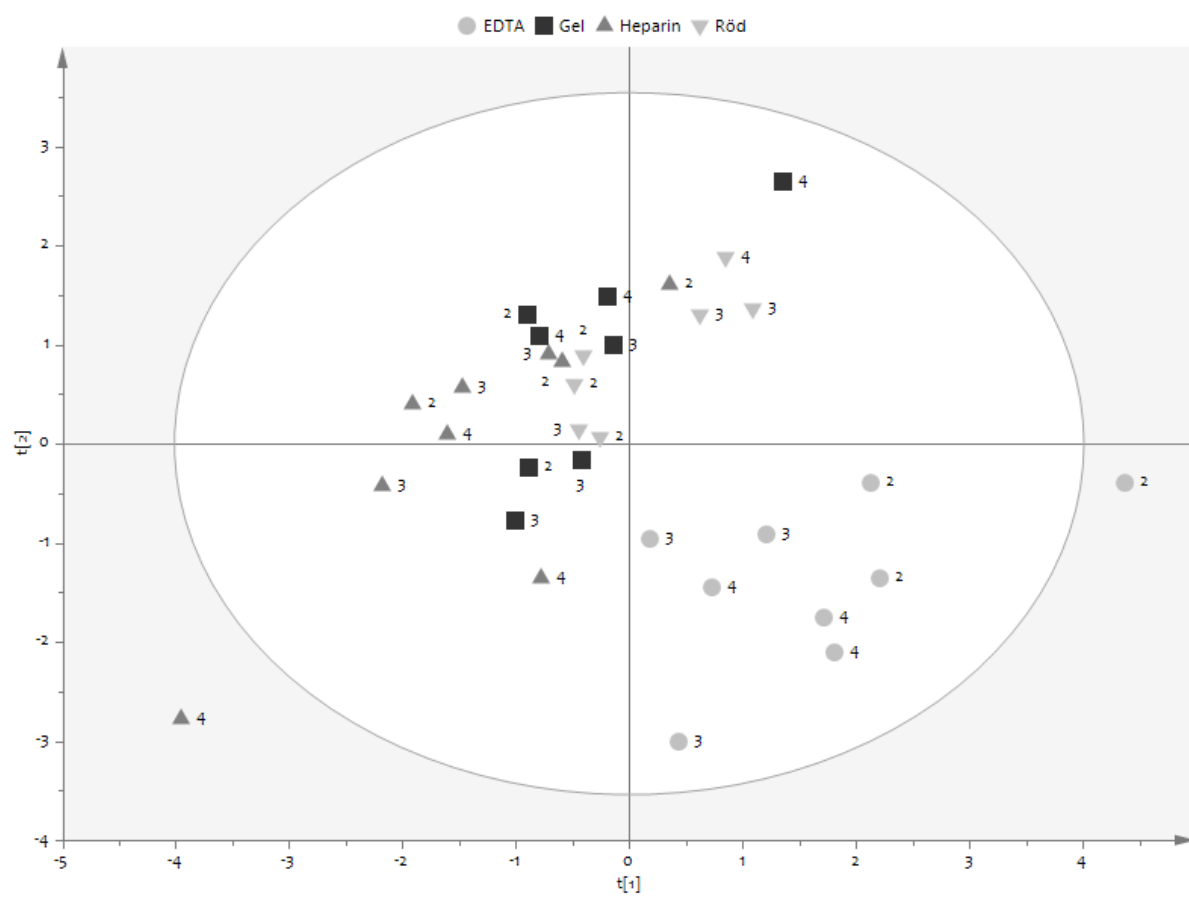

Supplement: S2 Fig — Labels show numbers of freeze-thaw cycles. Data points denote whether it is a serum sample (■gel and▼red) or a plasma sample (○EDTA and ▲heparin) (PDF) [file pone.0141738.s002.pdf]

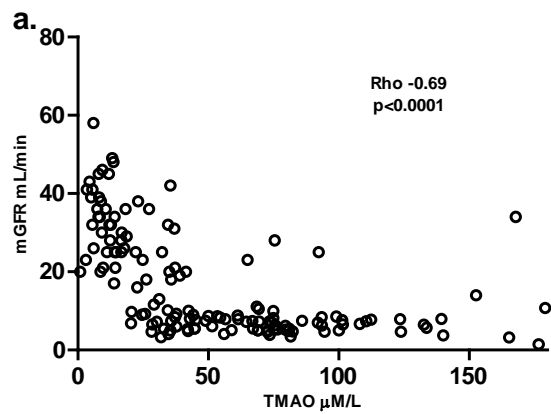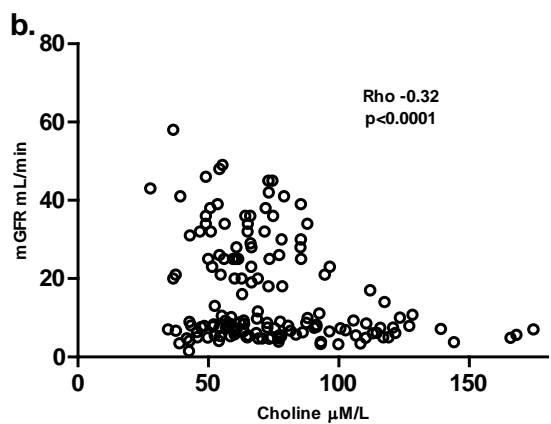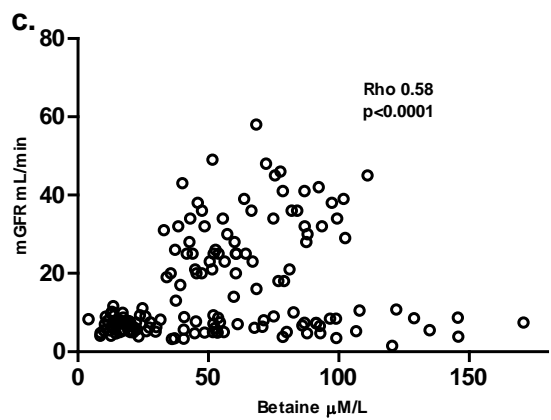

Supplement: S3 Fig — (PDF) [file pone.0141738.s003.pdf]

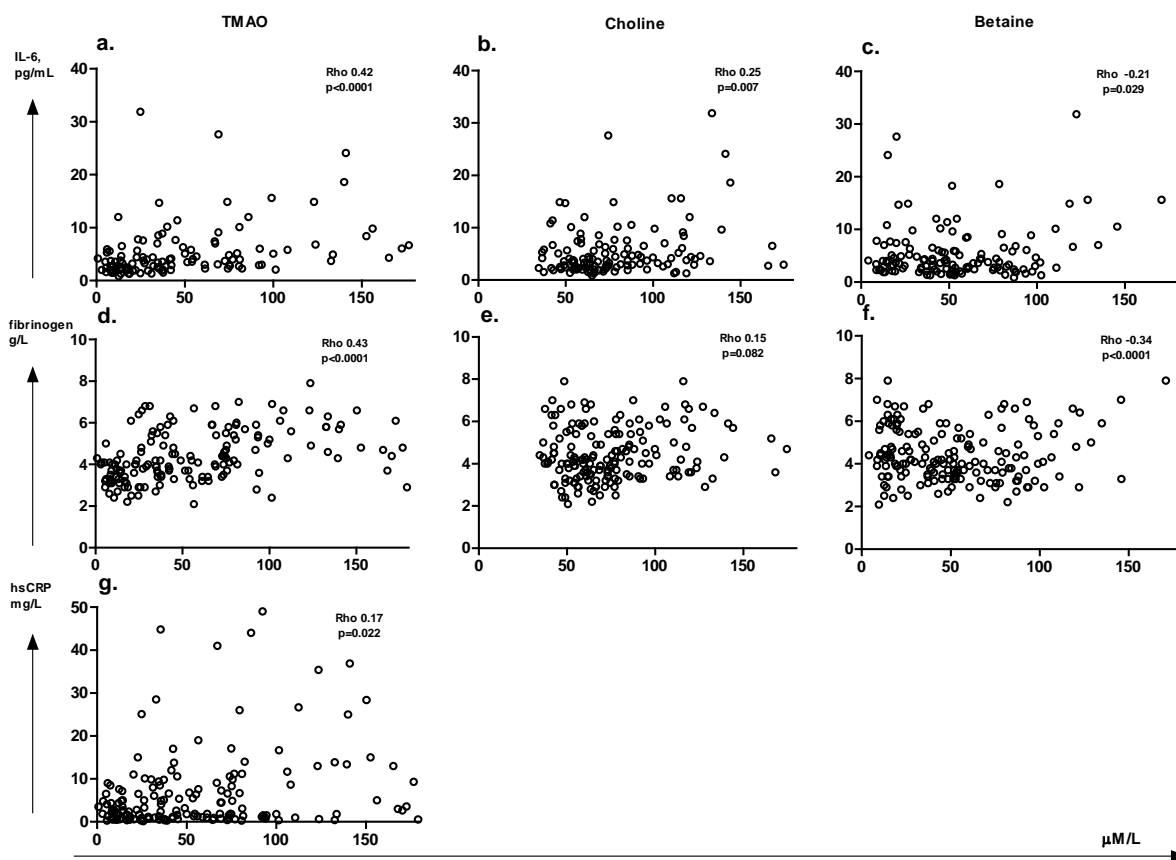

Supplement: S4 Fig — (PDF) [file pone.0141738.s004.pdf]
