# Supplementary material for: Serum Trimethylamine-N-Oxide Is Strongly Related to Renal Function and Predicts Outcome in Chronic Kidney Disease
Source: PLoS One. 2016 Jan 11;11(1):e0141738. doi: 10.1371/journal.pone.0141738 (PMC4709190; doi:10.1371/journal.pone.0141738)
Supplement: S1 Table — (PDF) [file pone.0141738.s005.pdf]

Person	Average ± SD	Person CV*(%)*	Intraday CV*(%)*	Interday CV*(%)*	Total CV*(%)*
I	0.94 ± 0.05	5.26	4.86	2.21	5.34
J	8.16 ± 0.12	1.14	1.07	1.00	1.47
K	9.35 ± 0.12	1.30	1.13	0.69	1.33
L	3.99 ± 0.07	1.80	1.79	0.13	1.80

Average, SD, CV(%) for person, within days, between days and total CV*(%)* are presented.

Person CV(%) was calculated by dividing the total standard deviation with the total average and multiplied by 100%.

Intraday CV(%) was calculated by taking the square root of the between days variance divided by the number of days, the root was then divided by the total average and multiplied by 100%.

Interday CV(%) was calculated by dividing the square root of the within group variance by the total average and multiplied by 100%.

Total CV(%) was calculated by taking the square root of the sum of the variance within groups and between days divided by the total average and multiplied by 100%.
